# Single-cell and bulk transcriptomics of the liver reveals potential targets of NASH with fibrosis

**DOI:** 10.1038/s41598-021-98806-y

**Published:** 2021-09-29

**Authors:** Zhong-Yi Wang, Adrian Keogh, Annick Waldt, Rachel Cuttat, Marilisa Neri, Shanshan Zhu, Sven Schuierer, Alexandra Ruchti, Christophe Crochemore, Judith Knehr, Julie Bastien, Iwona Ksiazek, Daniel Sánchez-Taltavull, Hui Ge, Jing Wu, Guglielmo Roma, Stephen B. Helliwell, Deborah Stroka, Florian Nigsch

**Affiliations:** 1 Novartis Institutes for BioMedical Research, 4056 Basel, Switzerland; 2 Visceral Surgery and Medicine, Inselspital, Bern University Hospital, Department for BioMedical Research, University of Bern, 3008 Bern, Switzerland; 3 China Novartis Institutes for BioMedical Research, Shanghai, 201203 China; 4 Present Address: Rejuveron Life Sciences AG, 8952 Schlieren, Switzerland

**Keywords:** Target identification, Data integration, Data processing, Functional clustering, Gene ontology, Gene regulatory networks, Genome informatics

## Abstract

Fibrosis is characterized by the excessive production of collagen and other extracellular matrix (ECM) components and represents a leading cause of morbidity and mortality worldwide. Previous studies of nonalcoholic steatohepatitis (NASH) with fibrosis were largely restricted to bulk transcriptome profiles. Thus, our understanding of this disease is limited by an incomplete characterization of liver cell types in general and hepatic stellate cells (HSCs) in particular, given that activated HSCs are the major hepatic fibrogenic cell population. To help fill this gap, we profiled 17,810 non-parenchymal cells derived from six healthy human livers. In conjunction with public single-cell data of fibrotic/cirrhotic human livers, these profiles enable the identification of potential intercellular signaling axes (e.g., ITGAV–LAMC1, TNFRSF11B–VWF and NOTCH2–DLL4) and master regulators (e.g., *RUNX1* and *CREB3L1*) responsible for the activation of HSCs during fibrogenesis. Bulk RNA-seq data of NASH patient livers and rodent models for liver fibrosis of diverse etiologies allowed us to evaluate the translatability of candidate therapeutic targets for NASH-related fibrosis. We identified 61 liver fibrosis-associated genes (e.g., *AEBP1, PRRX1* and *LARP6*) that may serve as a repertoire of translatable drug target candidates. Consistent with the above regulon results, gene regulatory network analysis allowed the identification of *CREB3L1* as a master regulator of many of the 61 genes. Together, this study highlights potential cell–cell interactions and master regulators that underlie HSC activation and reveals genes that may represent prospective hallmark signatures for liver fibrosis.

## Introduction

There has been a steady rise in the worldwide prevalence of nonalcoholic fatty liver disease (NAFLD)^1^. NAFLD and its advanced form nonalcoholic steatohepatitis (NASH) are the most common etiologies of hepatic fibrosis and represent a significant health burden^1^. As the largest internal organ in the human body, the liver performs many diverse functions that are essential for health, mainly carried out by hepatocytes, the parenchymal cells^2^. Liver non-parenchymal cells (NPCs) consisting of endothelial cells (ECs), hepatic stellate cells (HSCs), Kupffer cells (KCs), lymphocytes and cholangiocytes support hepatocyte function. Depending on the etiology of liver disease, HSCs and, to a lesser extent, portal fibroblasts and mesothelial cells (MCs) have long been closely linked to driving the development of hepatic fibrosis^3^. Under healthy conditions, HSCs maintain plasticity and respond to fluctuations in tissue physiology^4^. However, cytokines and growth factors secreted from adjacent or remote cells can trigger the trans-differentiation of quiescent HSCs (qHSCs) into fibrogenic activated HSCs (aHSCs). aHSCs produce excessive amounts of collagen and other extracellular matrix (ECM) components that impede the exchange of molecules between hepatocytes and the blood^5,6^.

In view of this, attenuation of fibrosis represents a major therapeutic goal, where antifibrotic agents can either inhibit fibrogenesis (ECM synthesis and deposition) or induce fibrolysis (removal of excessive ECM)^7^. Our understanding of the mechanisms underlying NASH with fibrosis has advanced steadily in the past two decades. Generally, anti-fibrotic therapies can be divided into agents that elicit anti-fibrotic effects by (1) hepatocyte protection, (2) immune modulation, or (3) inhibition of HSC activation and fibrotic scar evolution^8^. However, despite the enormous work and resources that have gone into the study of this disease and the fact that many mechanisms of liver fibrosis have been described^5,9^, there are currently no drugs approved specifically for NASH and liver fibrosis. Initial attempts targeting aHSCs directly through inhibition of tyrosine kinase receptors (e.g., PDGFRα/β) or TGF-β receptors were abandoned because of likely adverse reactions due to pleiotropic expression of these receptors^10^. To date, very few efforts focusing on inhibiting fibrogenesis have advanced into clinical trials. For example, simtuzumab, a humanized IgG4 anti-lysyl oxidase like 2 (LOXL2) monoclonal antibody was discontinued after a phase 2 clinical trial in NASH patients with liver fibrosis (clinicaltrials.gov—NCT01672879). Multiple factors may have contributed to this failure to clinically translate the overall positive preclinical results^11^. Another approach to promote stellate cell apoptosis through a siRNA targeting heat shock protein 47 (HSP47), a collagen 1 chaperone, was tested in phase 1 in patients with moderate to extensive fibrosis (clinicaltrials.gov—NCT02227459). More studies on collagen inhibitors are expected to start in the next years^8^. Fibrolysis-inducing strategies, including (1) upregulation of expression and/or activity of matrix metalloproteinases (MMPs) or (2) downregulation of expression and/or activity of tissue inhibitors of MMPs (TIMPs), also have promise^12^. However, no therapy that accelerates fibrolysis has reached clinical trials.

To achieve a broader understanding of fibrotic processes and deciphering the molecular mechanisms that accompany NASH-associated fibrosis, many transcriptomic studies in model organisms and humans have been performed^13–18^. Yet, these studies were largely restricted to sequencing RNA from mixed cell populations, heavily biased towards hepatocytes, which likely vary dependent upon the stage of disease. Therefore, despite the successful identification of many fibrosis-related genes and pathways^5^, these transcriptomic studies could not differentiate the common fibrogenic changes experienced across all liver cells from those that may be cell-type specific. Thus, cell-type specific gene expression studies of liver cell types from pathologic settings are crucial to uncover cellular crosstalk during HSC activation and to provide new therapeutic avenues.

The power and utility of single-cell RNA-sequencing (scRNA-seq) for gene-expression analyses have been shown in studies to identify previously unknown cell types and subtypes in normal and diseased liver^19,20^. To date, scRNA-seq has been conducted on whole liver or NPCs from humans^21–24^ and mice^25–30^. However, thus far only one human study has investigated hepatic injury in the context of cirrhosis^22^.

In this study, we isolated and profiled NPCs from healthy human livers, and integrated public scRNA-seq data derived from fibrotic/cirrhotic human livers. We also generated time-course RNA-seq data for both thioacetamide (TAA) and bile duct ligation (BDL)-induced liver fibrosis rat models. Additionally, we reanalyzed public transcriptomic data from in vivo and in vitro mouse models of liver fibrosis to determine the key genes associated with HSC activation in liver fibrosis of different etiologies.

## Results

### Single-cell profiling and unbiased clustering of human liver cells

To examine the heterogeneity and dynamic crosstalk of liver NPCs, we generated scRNA-seq data for human liver using the 10× Genomics Chromium platform. Since hepatocytes constitute approximately 60% of the cells in the liver^2^, to enrich for NPCs we adopted a method^31^ to remove hepatocytes prior to sequencing library preparation (Fig. 1a, “Methods”).

**﻿Figure 1:**
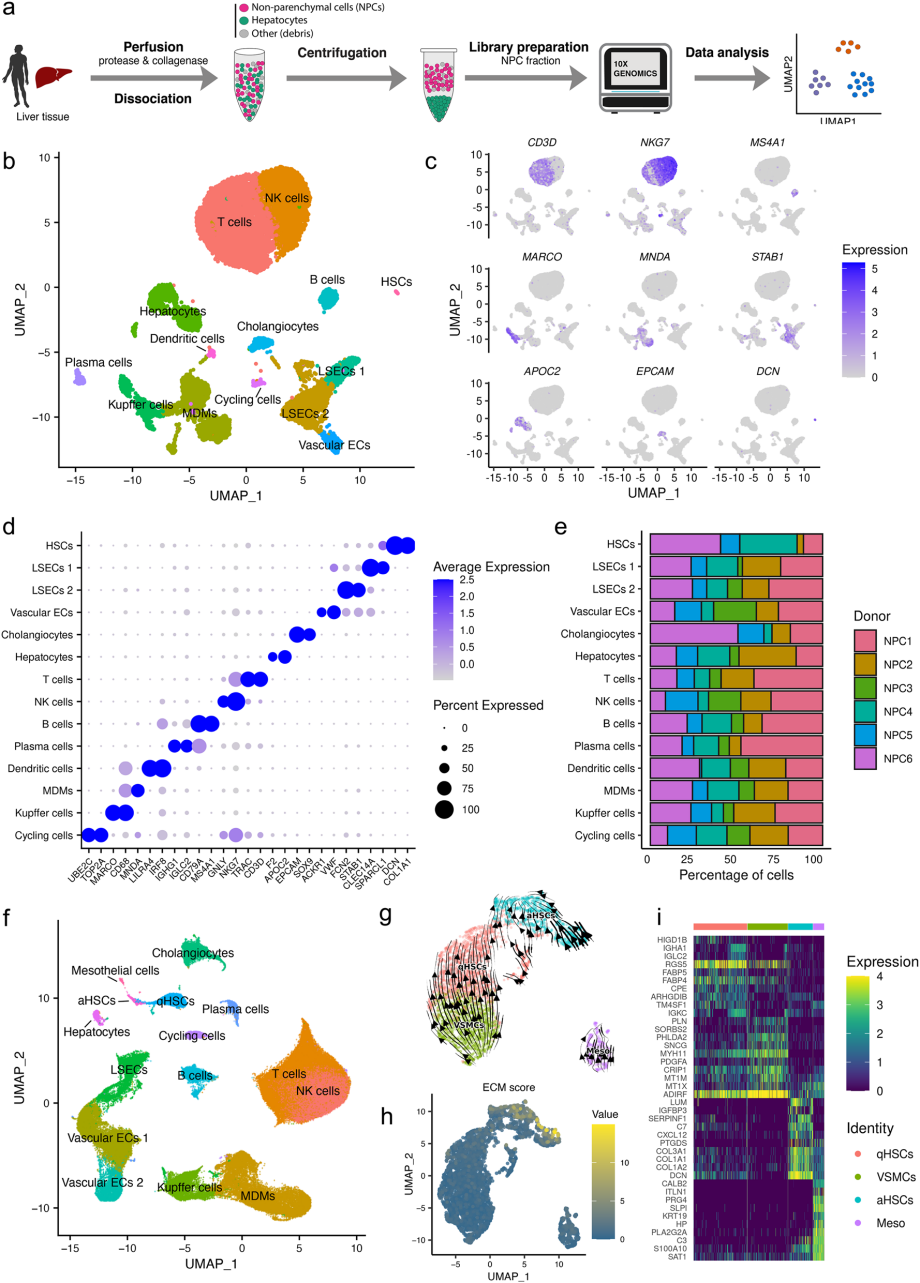
Generation of scRNA-seq data for human liver non-parenchymal cells. (**a**) Workflow of sample preparation, sequencing and bioinformatic analysis. (**b**) UMAP visualization of single cells profiled in this study. Each dot represents a cell that is color-coded by cell type. HSCs, hepatic stellate cells; LSECs, liver sinusoidal endothelial cells; Vascular ECs, vascular endothelial cells; MDMs, monocyte-derived macrophages; NK cells, natural killer cells. (**c**) Representative gene expression and distribution of known marker genes for each population in UMAP plots. Normalized expression values are shown. (**d**) Dotplot displaying the top two marker genes for each cell type identified. Size of the dot represents proportion of the cell population that expresses each gene. Color indicates level of expression. (**e**) Barplot representing the relative contribution of cells from each donor for each cell type. NPC, non-parenchymal cells derived from healthy liver. (**f**) UMAP visualization of single cells profiled in this study together with human liver cells integrated from Ramachandran et al. (2019). (**g**) UMAP visualization of mesenchymal cells. The direction of cell differentiation inferred from estimated RNA velocities are plotted as streamlines on the UMAP. (**h**) UMAP visualization of mesenchymal cells. Cells colored by ECM score. (**i**) Heatmap showing the top 10 differentially expressed genes for each mesenchymal cell type. VSMCs, vascular smooth muscle cells; Meso, mesothelial cells.

We isolated and profiled 17,810 cells from six healthy human livers. After removing low-quality cells, a total of 15,299 cells (1449–4821 per sample) were retained for further analysis. A median of 1141 genes per cell was detected. To identify cells with distinct lineage identities and transcriptional states, we performed unbiased clustering on the cells using the Seurat R package^32^. NPCs were represented by a total of 13 distinct cell lineages (Fig. 1b), which correspond to hepatic stellate cells (HSCs; *COL1A1*^+^*DCN*^+^), liver sinusoidal endothelial cells (LSECs 1: zone 1, *SPARCL1*^+^*CLEC14A*^+^; LSECs 2: zone 2 and 3, *STAB1*^+^*FCN2*^+^), vascular ECs (*VWF*^+^*ACKR1*^+^), cholangiocytes (*SOX9*^+^*EPCAM*^+^), T cells (*CD3D*^+^*TRAC*^+^), natural killer (NK) cells (*NKG7*^+^*GNLY*^+^), B cells (*MS4A1*^+^*CD79A*^+^), plasma cells (*IGLC2*^+^*IGHG1*^+^), dendritic cells (*IRF8*^+^*LILRA4*^+^), monocyte-derived macrophages (MDMs; *CD68*^+^*MNDA*^+^), Kupffer cells (*CD68*^+^*MARCO*^+^) and cycling cells (*TOP2A*^+^*UBE2C*^+^) (Fig. 1c,d). Because hepatocytes were not fully removed during the centrifugation step, there was also a small cluster of hepatocytes (*APOC2*^+^*F2*^+^) (Fig. 1b–d). All 14 cell types contained cells derived from each of the six liver donors (Fig. 1e). We also further examined our cell type annotations with SingleR^33^—a computational framework that compares the transcriptomic profile of each single cell to reference datasets to determine cellular identity (Supplementary Fig. 1).

To gain insight into the activation of HSCs, we aggregated 157,619 cells from four healthy and importantly also three cirrhotic human livers of a public dataset^22^. A total of 175,429 cells were then analyzed (“Methods”). 20 populations and 15 distinct cell lineages were subsequently identified (Fig. 1f, Supplementary Fig. 2).

Since in the current study the main focus is mesenchymal cells (also known as ECM-producing cells, including HSCs, portal fibroblasts and perivascular cells), we carried out subclustering of these populations and performed RNA velocity analysis (Fig. 1g, “Methods”). This analysis revealed that the robustness of developmental trajectories from *RGS5*-expressing qHSCs to *COL1A1*-expressing aHSCs was supported. ECM scores, calculated based on the expression values of co-expressed ECM-related genes (“Methods”), also demonstrated a clear shift towards higher ECM expression in mesenchymal cells from the cirrhotic patient samples, with aHSCs exhibiting the highest ECM expression (Fig. 1h). We then identified the marker genes for each cell type by performing differential gene expression analysis (Fig. 1i). Among the most differentially expressed genes for aHSCs there were well-known fibrosis markers, e.g., *COL1A1*, *COL1A2, DCN* and *LUM*.

### Identification of liver fibrosis-related intercellular communication

**Figure 2:**
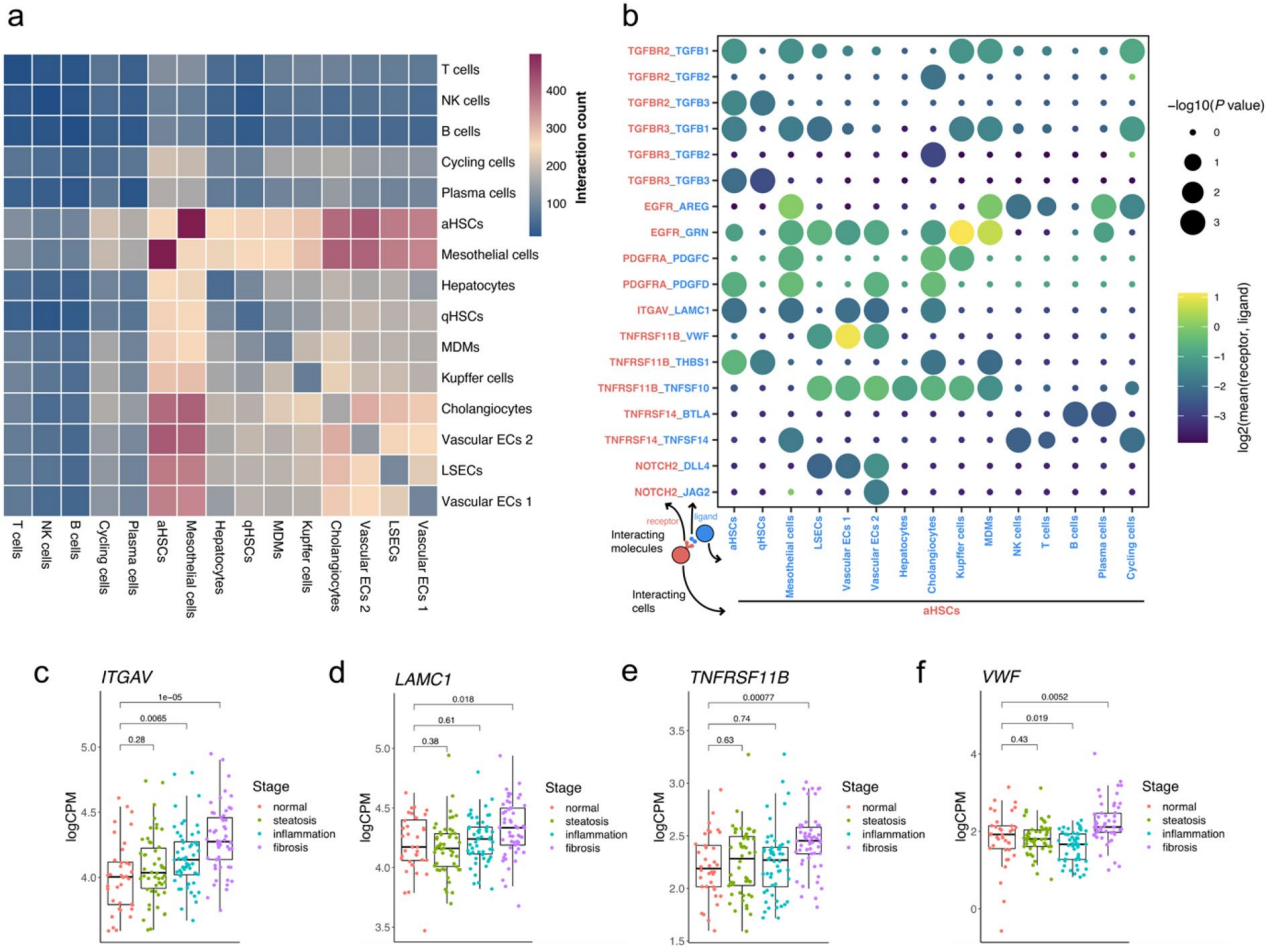
Intercellular communications in the sinusoidal signaling niche. (**a**) Heatmap showing the number of potential ligand-receptor pairs between any two liver cell types predicted by CellphoneDB. (**b**) Dotplot displaying putative ligand-receptor interactions between aHSCs and other cell types. Dot size represents statistical significance of the indicated interactions. Dot color indicates the average expression level (log_2_-transformed) of the receptor from aHSCs and the ligand from another cell type. (**c**–**f**) Gene expression of *ITGAV*, *LAMC1*, *TNFRSF11B*, and *VWF* at progressive disease stages. The RNA-seq data were downloaded and reanalyzed from Gerhard et al. (2018). Mann–Whitney *U* tests (two-sided) were performed for statistical comparisons. logCPM, log-transformed counts per million.

Cell–cell signaling is critical in cell development, tissue homeostasis and disease development^34^. HSC activation is a process that is coordinated by intercellular signaling effectors that modulate the activity of downstream gene regulatory networks^5,6^. By leveraging the transcriptional profiles of each cell population, we built a comprehensive intercellular network of potential ligand-receptor interactions between different cell types using CellPhoneDB^35^ with curated known ligand-receptor pairs from databases^35^ and literature^36^ (Fig. 2a). The number of potential interactions between aHSCs and other cell types is significantly larger than that between qHSCs and other cell types (paired Student's t-test, *P* = 9.12e-7; Fig. 2a). We focused specifically on interactions that involve HSCs and used the interaction pairs between qHSCs and other cells as references to pinpoint aHSC-specific interactions that may represent potential key mediators involved in the initiation or perpetuation of liver fibrosis. Figure 2b shows selected significant (*P* < 0.05) ligand-receptor pairs that are specific to aHSCs.

The most significant specific interactions were predicted to happen in the sinusoidal signaling niche, where HSCs are in close proximity to KCs/MDMs, LSECs and vascular ECs. The transforming growth factor (TGF)-β signaling pathway is associated with liver fibrosis and HSC activation^37^ and was indeed present in our results (Fig. 2b). Ligands potentially binding to epidermal growth factor receptor (EGFR) on aHSCs include amphiregulin (AREG) and progranulin (GRN) (Fig. 2b). Robust expression of *AREG* in MDMs and other immune cells raises the possibility of EGFR signaling. Consistently, the EGFR–AREG signaling axis has indeed been implicated in HSC fibrogenic activity, and *AREG* was upregulated in murine and human NASH^38^. KCs and MDMs may also activate HSCs by secreting GRN that modulates EGFR signaling, and GRN was shown to function as a HSC survival signal by stimulating EGFR signaling through ephrin receptor A2^39^. Erlotinib could attenuate EGFR phosphorylation in HSCs and subsequently reduce the total number of aHSCs^40^. PDGFC secreted from KCs and cholangiocytes could bind to the platelet-derived growth factor receptor α (PDGFRα) of HSCs and then trigger the PDGF signaling pathway (Fig. 2b). This is in line with previous studies showing that PDGF signaling pathway exerts persistent activation in response to a variety of stimuli and facilitates HSC activation and fibrosis progression^41^. HSC activation may also be amplified through autocrine signaling (PDGFD–PDGFRα) (Fig. 2b). Interestingly, PDGF-C and -D levels increased during the trans-differentiation of qHSCs into aHSCs and persisted upon activation perpetuation, suggesting a role of these subunits in the late phase of fibrogenesis^42^.

Previous studies suggested that integrin-alpha V (ITGAV) may serve as a master driver of fibrosis in multiple mouse organs^43^. Of the potential alpha subunits, only ITGAV has been shown to be critical for binding and activating TGF-β, which plays a critical role in HSC activation^44^. Laminin subunit gamma 1 (LAMC1) secreted from neighboring cells could interact with ITGAV to trigger HSC activation (Fig. 2b), which is in agreement with our observation of increased RNA expression for both genes from healthy to fibrotic livers (Fig. 2c,d). TNF receptor superfamily member 11B (TNFRSF11B) on the surface of HSCs may be modulated by ligands including von Willebrand factor (VWF), thrombospondin 1 (THBS1) and TNFSF10 that are secreted from ECs, epithelial cells and KCs/MDMs (Fig. 2b). The TNFRSF11B–VWF signaling axis might serve as a novel interaction that could play a role in HSC activation, and VWF deficiency has been demonstrated to attenuate chronic carbon tetrachloride (CCl_4_)-induced liver fibrosis^45^. This is further bolstered by the gene expression comparisons of both genes in healthy and fibrotic human livers (Fig. 2e,f). Furthermore, B- and T-lymphocyte attenuator (BTLA) and TNFSF14 secreted by immune cells potentially interact with TNFRSF14, emphasizing the proinflammatory and proliferative roles aHSCs may play at the site of injury (Fig. 2b). Consistent with the literature^46^, we also found NOTCH signaling pathway to be involved in fibrogenesis, highlighting two potential interaction pairs between NOTCH2 on aHSCs and ligands DLL4 and JAG2 expressed in ECs (Fig. 2b). A recent study showed that *DLL4* knockdown alleviated LSEC capillarization and provided protection against CCl_4_-induced fibrosis^47^.

### Single-cell regulatory network inference for liver mesenchymal cells

**Figure 3:**
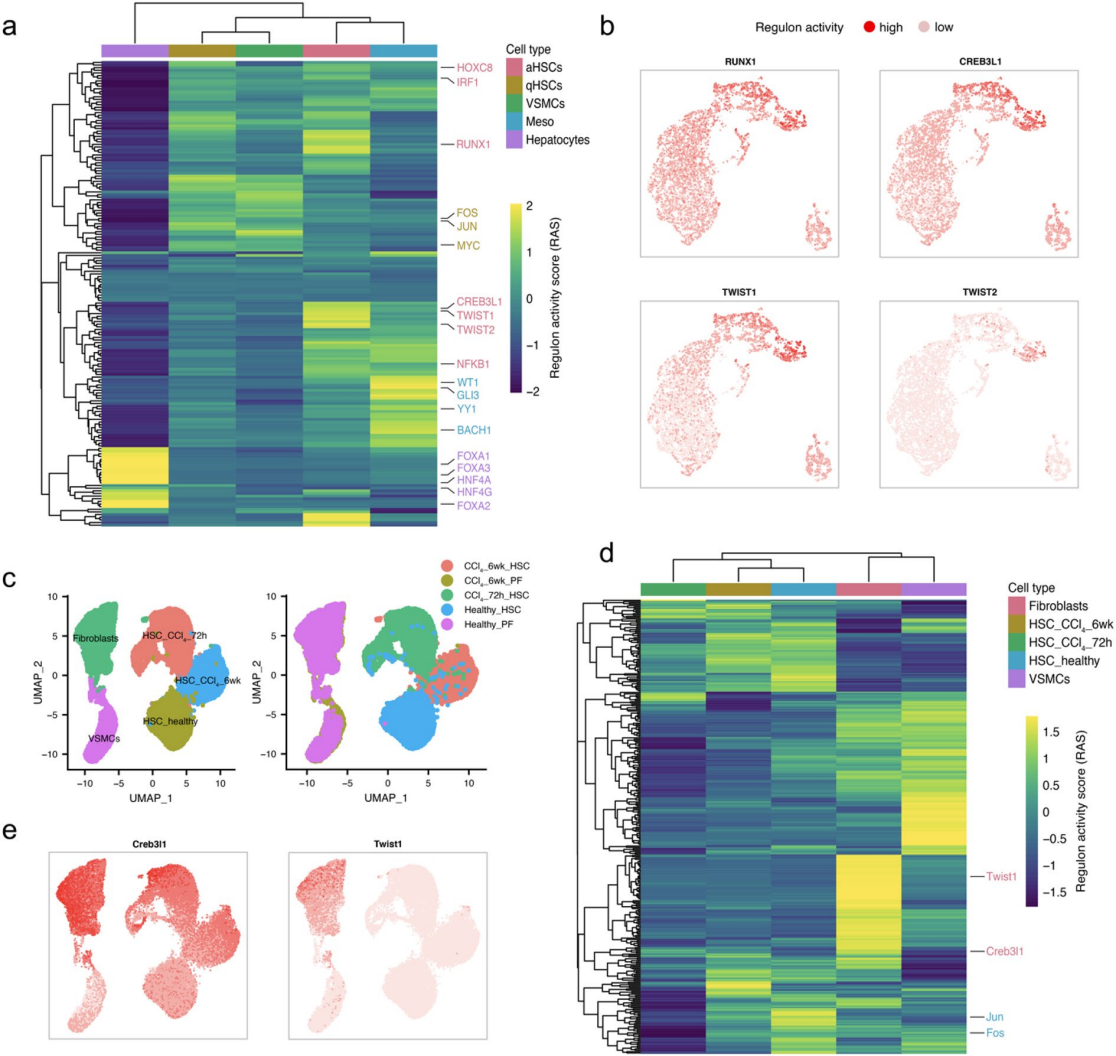
Cell type-specific gene regulatory networks (regulons) of liver mesenchymal cells. (**a**) Heatmap of the inferred regulons for human liver mesenchymal cells. (**b**) Regulon activity-based UMAP colored according to the regulon activity of CREB3L1, RUNX1, TWIST1 and TWIST2 showing the cell type specificity of regulons. (**c**) UMAP embedding of mouse liver mesenchymal cells from uninjured and fibrotic (up to 6 weeks of CCl_4_ treatment) mouse livers retrieved from Dobie et al. (2019). Left, cells colored by cell type/state. Right, cells colored by condition/cell origin. (**d**) Heatmap of the inferred regulons for mouse liver mesenchymal cells. (**e**) Regulon activity-based UMAP colored by the regulon activity of Creb3l1 and Twist1 showing the cell type specificity of regulons.

Gene regulatory networks define and maintain cell-type specific identity, which in turn determines cellular function. For this analysis, we specifically focused on the mesenchymal cells (Fig. 1g–i). To identify the master regulators for each cell, we applied the SCENIC pipeline^48^. Briefly, SCENIC predicts the transcription factors (TFs) alongside their candidate target genes, which are jointly called regulons. Three major steps are involved, namely, co-expression analysis, target gene motif enrichment analysis, and regulon activity evaluation. To examine whether this approach is effective, we included hepatocytes as their core gene regulatory networks have been well-characterized^49^. The analysis yielded cell-type specific regulons along with their respective regulon activity scores (RAS). The RAS were then averaged across cells for each cell type. Importantly, the regulon activity dendrogram for the 176 significant regulons containing in total 11,920 target protein-coding genes in Fig. 3a matches well with the clustering defined using all robustly expressed (18,771) protein-coding genes (Supplementary Fig. 3). It has indeed been suggested to use cell type-specific core regulatory complexes (CoRC) to define cell types^50^. Consistent with the literature^49^, SCENIC successfully identified known hepatocyte-specific regulators, including *HNF4A/G* and *FOXA1/2/3*, as the top regulons for hepatocytes (Fig. 3a).

The success of this approach in identifying critical hepatocyte regulators allowed us to examinate aHSC-specific regulons with confidence. Notably, we found that regulon RUNX1 was highly active in aHSCs but low in qHSCs and other cell types (Fig. 3a,b), suggesting that it is a potential master regulator of HSC activation. Indeed, *Runx1* was suggested to play important roles in NASH-associated HSC activation in mice^17^, and increased expression of *RUNX1* in human liver biopsies correlated with NASH activity score in patients with NASH^51^. Interestingly, *Runx1* was also identified as one of the top TFs in lipofibroblasts of fibrotic mouse lungs^52^.

*CREB3L1*, also known as OASIS, was identified as another potential master regulator of HSC activation (Fig. 3a,b), as its predicted target genes include known liver fibrosis-associated genes such as *COL1A1* and *LOX*. This observation is in agreement with a previous finding that CREB3L1-deficient mice exhibited severe osteopenia caused by a decrease in the expression of *COL1A1* in the bone matrix^53^. Another study revealed that inhibition of regulated intramembrane proteolysis of CREB3L1 could prevent excess deposition of collagen in some fibrotic diseases^54^. By leveraging a recent transcriptomic study on human NASH^13^, we showed that the expression of *CREB3L1* increases as the disease progresses from healthy to fibrotic liver (Supplementary Fig. 4). Reanalysis of a mouse single-cell dataset^25^ showed specifically high activity in aHSCs for the Creb3l1 regulon (Fig. 3c–e). In line with a recent study^55^, other aHSC-associated regulators (i.e., *IRF1* and *NFKB1*) were also observed in our results.

Furthermore, *CREB3L1*, *HOXC8*, *TWIST1* and *TWIST2* that were identified in this study are also among the TFs that were shared in fibroblasts of different organ origins^56^ (Fig. 3a,b). While the involvement of *HOXC8* in fibrogenesis still remains unexplored, the role of *TWIST1* in various fibrotic diseases has been gradually revealed in recent years^57^. Activation of *TWIST* transcription by BRG1, a chromatin remodeling protein, was suggested to contribute to liver fibrosis in mice and a TWIST1 inhibitor (harmine) exerted anti-fibrogenic effects^58^. Additionally, we identified specific regulons for other cell types, for example FOS, JUN and MYC for qHSCs, BACH1, GLI3, WT1 and YY1 for MCs (Fig. 3a). Two full lists of regulons and their scaled activity for each cell type can be found in Supplementary Tables 1 and 2, for human and mouse, respectively.

### Comparison of stellate cells between liver and pancreas

**Figure 4:**
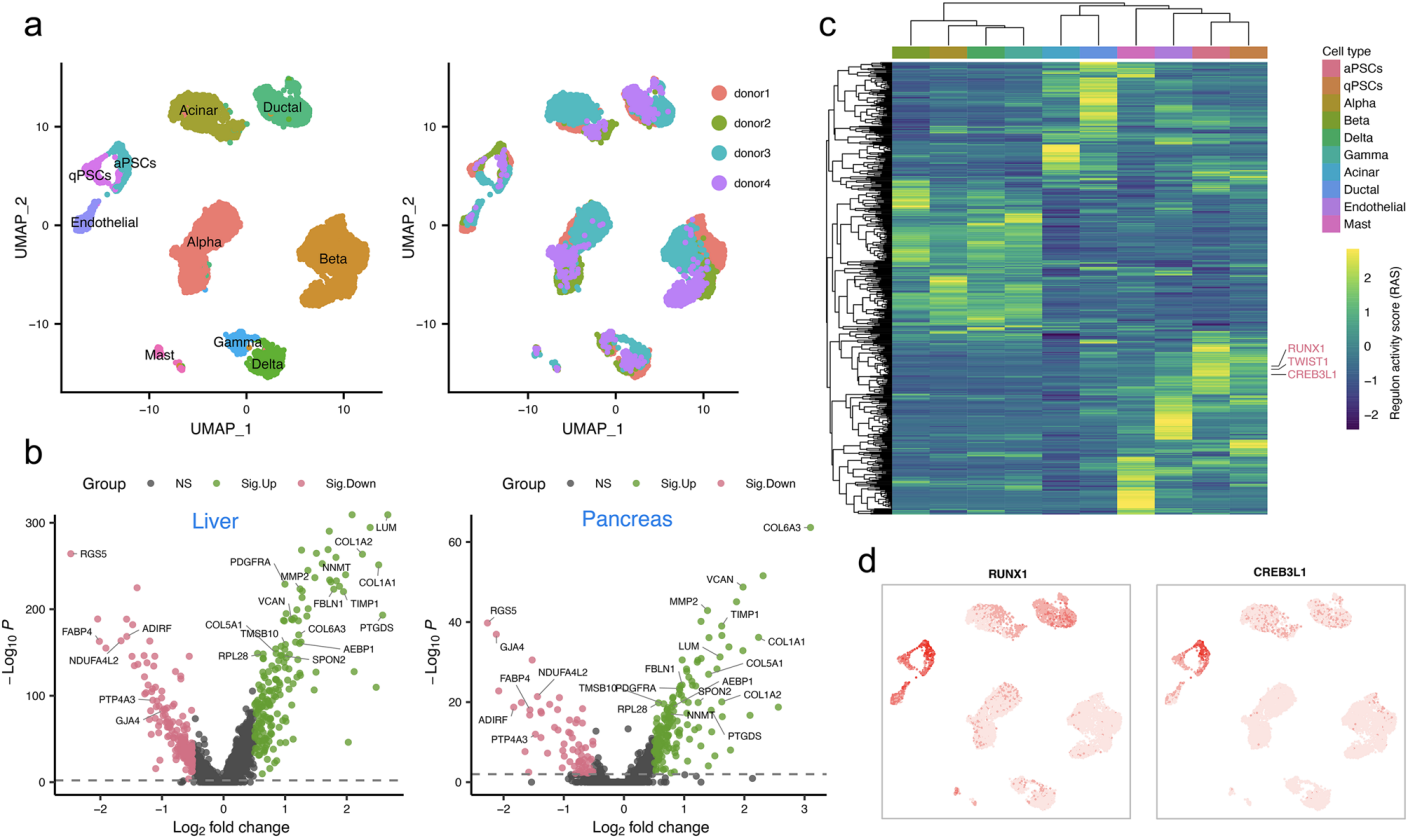
Comparison of expression patterns between hepatic and pancreatic stellate cells. (**a**) UMAP embedding of human pancreatic cells retrieved from Baron et al. (2016). Left, cells colored by cell type. Right, cells colored by donor. qPSCs, quiescent PSCs; aPSCs, activated PSCs. (**b**) Volcano plot of differential gene expression analysis between activated and quiescent stellate cells for liver (left) and pancreas (right). Significantly upregulated (Sig.Up) and downregulated (Sig.Down) genes in activated stellate cells are shown in green and pink, respectively. 16 and 6 genes shared among the top 50 up- or down-regulated genes between liver and pancreas are labelled, respectively. (**c**) Heatmap of the inferred regulons for human pancreatic cells. (**d**) Regulon activity based UMAP colored according to the regulon activity of CREB3L1 and RUNX1 showing the cell type specificity of regulons.

Pancreatic stellate cells (PSCs) share many morphological and functional characteristics with HSCs; they are thus presumed to share a common origin^59^. Despite the established role of stellate cells in fibrogenesis, there is currently still a lack of genomic comparison between stellate cells from liver and pancreas. A microarray-based transcriptomic analysis comparing HSCs to PSCs revealed that these cells are highly similar^60^. This analysis, however, appeared to be rather limited. scRNA-seq enables the comparison between HSCs and PSCs at unprecedented resolution to uncover overlapping and distinct gene expression features. To this end, we retrieved and reanalyzed a public human pancreas scRNA-seq dataset^61^, and identified two clusters that correspond to quiescent and activated PSCs (Fig. 4a). We next compared the up- and down-regulated genes in aHSCs and aPSCs (Fig. 4b). Many signature genes were common for stellate cells in both organs, for example *RGS5*, *FABP4* and *ADIRF* for quiescent stellate cells and *AEBP1*, *COL1A1*, *COL1A2*, *LUM*, *TIMP1* and *VCAN* for activated stellate cells (Fig. 4b).

Another question of interest is whether cells of the same type from different organs may share gene regulatory circuitries. To this end, we also performed the SCENIC analysis on all pancreatic cells. Indeed, we found that the stellate cells from both organs share certain regulon activities (Fig. 4c). Consistent with the findings for aHSCs, the regulon activities of RUNX1, CREB3L1 and TWIST1 were significantly higher in aPSCs in comparison to qPSCs (Fig. 4c,d). A full list of regulons and their scaled activity for each cell type can be found in Supplementary Table 3.

### Evaluating translatability of liver fibrosis-associated genes

**Figure 5:**
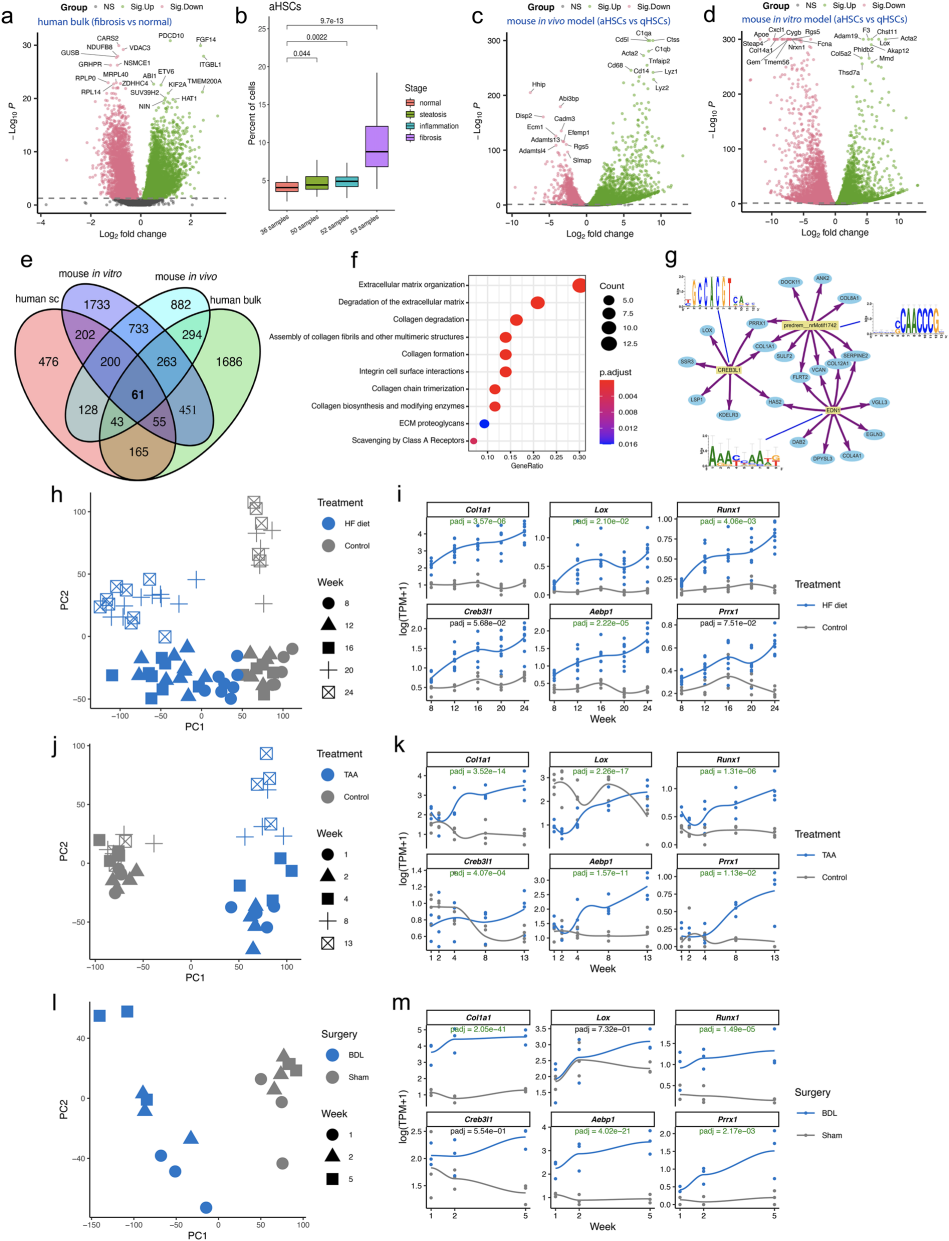
Meta-analysis of existing RNA-sequencing data sets for NASH and liver fibrosis. (**a**) Differential gene expression analysis between fibrotic and healthy (as reference) livers. Only the top 10 up- or down-regulated genes are labelled with gene symbols. Significantly upregulated (Sig.Up) and downregulated (Sig.Down) genes are shown in green and pink, respectively. (**b**) Computational deconvolution of bulk RNA-seq data of 191 human liver samples (normal, 36; steatosis, 50; inflammation, 52; fibrosis, 53). The RNA-seq data were retrieved from Gerhard et al. (2018). Mann–Whitney *U* tests (two-sided) were performed for statistical pairwise comparisons. (**c**) Differential gene expression analysis between aHSCs and qHSCs (as reference) isolated from healthy and fibrotic (CCl_4_) mouse livers. The RNA-seq data were retrieved from Marcher et al. (2019). Significantly upregulated (Sig.Up) and downregulated (Sig.Down) genes are shown in green and pink, respectively. Only the top 10 up- or down-regulated genes are labelled with gene symbols. (**d**) Differential gene expression analysis between aHSCs and qHSCs (as reference)*.* Healthy liver-derived qHSCs were activated on plastic in vitro. The RNA-seq data were retrieved from Marcher et al. (2019). Significantly upregulated (Sig.Up) and downregulated (Sig.Down) genes are shown in green and pink, respectively. Only the top 10 up- or down-regulated genes are labelled with gene symbols. (**e**) Venn diagram showing all possible logical relations between three sets of up-regulated genes in aHSCs compared with qHSCs, and a set of up-regulated genes in bulk human fibrotic livers in comparison to healthy controls. Only the 15,252 protein-coding one-to-one orthologs for human, mouse, and rat were considered in this analysis (Supplementary Table 6). (**f**) Functional enrichment analysis for the 61 shared upregulated genes. (**g**) Regulatory gene network of the top three regulators (motifs) and their corresponding target genes among the 61 genes. (**h**–**m**) Contextualization of candidate therapeutic targets for liver fibrosis with time-course RNA-seq data. (**h**,**i**) High-fat (HF) diet-induced NASH mouse model; (**j**,**k**) Thioacetamide (TAA)-induced liver fibrosis rat model; (**l**,**m**) Bile duct ligation (BDL)-induced liver fibrosis rat model. Left, PCA of the samples studied. Right, expression levels of the genes of interest. TPM, transcripts per million. Adjusted p-values < 0.05 are colored in green.

We next sought to explore our findings with a focus on translatability. The need for effective and safe therapy has spurred the development of in vitro and in vivo models to study the relevance of specific genes for the development and progression of NASH and to evaluate potential therapeutic agents prior to human studies^7,62^. By leveraging bulk RNA-seq data of NASH patient livers and rodent models for liver fibrosis of diverse etiologies, we were able to evaluate the translatability of candidate therapeutic targets for NASH with fibrosis. An analysis of a human NASH-related RNA-seq dataset^13^ confirmed that many of the aHSC-specific genes were indeed upregulated in fibrotic compared to healthy livers (Fig. 5a). Consistent with this finding, computational deconvolution of this dataset showed an upward trend in the proportion of aHSCs over the course of disease development (Fig. 5b). Next, to evaluate the translatability of potential novel therapeutic targets that were identified based on human data, we integrated public RNA-seq datasets from mouse models of CCl_4_-induced fibrosis and in vitro HSC activation^17^. For the in vivo mouse model, qHSCs and aHSCs were sorted separately from healthy and fibrotic livers after 8 weeks of CCl_4_ treatment. For the in vitro mouse model, healthy liver-derived primary qHSCs were cultured and activated on plastic for 12 days. Differential gene expression analyses between aHSCs and qHSCs were subsequently carried out (Fig. 5c,d). In total, we identified 61 significantly upregulated and 13 downregulated genes that behave similarly in human aHSCs and fibrotic liver tissues, as well as in mouse aHSCs from in vivo and in vitro models of liver fibrosis (Fig. 5e, Supplementary Tables 4 and 5).

We then focused specifically on the 61 significantly upregulated genes. Functional enrichment analysis revealed pathways where the 61 genes were overrepresented, with the top 10 most enriched pathways including those related to ECM organization, collagen formation and degradation, and integrin cell surface interactions (Fig. 5f). Together, this highlights the central roles of ECM and integrins in the development of liver fibrosis, regardless of the etiological origin of the disease. A gene regulatory network was also built for the 61 DEGs to better understand potential coregulation by master TFs responsible for the development of fibrosis. Target genes, the top three motifs and corresponding TFs (if information available) are shown in Fig. 5g. Consistent with the above regulon results, *CREB3L1* is again identified as a master regulator of liver fibrosis-related genes.

We next examined individual genes. Many of these genes, including *COL1A1*, *LOX*, *MMP14*, *TGFB3*, *TIMP1* and *VCAN*, are known markers for liver fibrosis. Other genes such as *AEBP1*, *PRRX1* and *LARP6* have also been implicated in liver fibrogenesis and could serve as potential drug targets for NASH with fibrosis. Noteworthily, *AEBP1* and *PRRX1* are the only two TF genes among the 61 genes. The expression of *AEBP1* was significantly elevated in fibrotic human liver compared to that with lobular inflammation, steatosis, and healthy liver, and increased with worsening fibrosis in NASH patients^63^. *AEBP1* is also among the most significantly upregulated genes in aPSCs compared with qPSCs (Fig. 4b). Interestingly, *Aebp1* was specifically expressed in the *Col14a1*-producing subpopulation of myofibroblasts in murine fibrotic lungs^52^. Using rodent models of liver fibrosis, a previous study uncovered a critical role of *Prrx1* in PDGF-dependent HSC migration, and an adenoviral-mediated *Prrx1* short hairpin RNA (shRNA) administration reduced thioacetamide (TAA)-and CCl_4_-induced liver fibrosis^64^. Consistently, *PRRX1* was recently identified to be involved in idiopathic pulmonary fibrosis, and inhibition of PRRX1 activity was sufficient to alleviate the development of pulmonary fibrosis^65^. As fibrosis is characterized by excessive accumulation of type I collagen, binding of LARP6 to the 5’ stem-loop structure of collagen mRNAs is a crucial step for collagen translation^66^. This makes *LARP6* a promising target for liver fibrosis, and a recent high throughput screen has yielded a compound (C9) that represents the first LARP6 inhibitor with significant antifibrotic activity^67^.

To further explore those genes that we have shown to be involved in liver fibrosis-associated pathways and gene regulatory circuitries, we leveraged a mouse time-course dataset of high fat (HF) diet-induced NASH^16^. Additionally, we generated time-course RNA-seq data for rat models of TAA- and BDL-induced liver fibrosis. Consistently, the expression of two well-known liver fibrosis-associated genes (e.g., *Col1a1* and *Lox*) among the 61 shared liver fibrosis-related genes and four TF genes of interest (e.g., *Runx1*, *Creb3l1*, *Aebp1* and *Prrx1*) showed an upward trend as disease worsens across all three rodent models (Fig. 5h–m), further confirming their translatability across human, mouse and rat for future drug discovery endeavors.

## Discussion

Fibrosis is a common sequela following organ injury, and HSC activation plays a pivotal role in liver fibrogenesis of different etiologies. Although activated HSCs represent an attractive target for antifibrotic therapy, the molecular mechanisms underlying the activation of HSCs remain poorly characterized. In this study, we performed scRNA-seq to determine the transcriptional dynamics of non-parenchymal cells of healthy human livers, and a subsequent integrative analysis of publicly available scRNA-seq data of fibrotic/cirrhotic livers enabled a deep dive into the mesenchyme, and HSCs in particular.

RNA velocity analysis revealed a clear cell state transition from *RGS5*-expressing HSCs to myofibroblast-like aHSCs that express ECM proteins and profibrotic mediators. Our analyses subsequently uncovered underlying cell–cell interactions and master regulons that potentially drive the activation of HSCs. Immune cells, epithelial cells (i.e., hepatocytes and cholangiocytes) and endothelial cells that dwell within the fibrotic niche communicate via secreting ligands that can potentially bind to specific cell surface receptors of HSCs to trigger the downstream signaling pathways that lead to the expression of liver fibrosis-associated genes. We highlighted three barely-characterized or entirely novel interactions (ITGAV–LAMC1, TNFRSF11B–VWF and NOTCH2–DLL4) that could lead to HSC activation or proliferation. For example, apart from our finding of an increased expression of both *TNFRSF11B* and *VWF* in fibrotic versus healthy livers (Fig. 2e,f), *VWF* deficiency was also previously demonstrated to attenuate chronic CCl_4_-induced liver fibrosis^45^. This suggests that inhibition of VWF may be a clinically useful anti-fibrotic therapy resulting from a decrease in TNFRSF11B–VWF signaling. Our further analysis also revealed regulons that are potentially responsible for HSC activation in both humans and mice, such as CREB3L1 and TWIST1. The comparison of hepatic and pancreatic stellate cells points to shared regulatory mechanisms across different organs, thereby providing novel avenues for therapeutic target selection: among the liver fibrosis-associated regulons CREB3L1 was shared not only between species (i.e., human and mouse) but also between human organs (i.e., liver and pancreas).

Animal models are indispensable tools to study the cellular and molecular mechanisms of liver fibrosis and to develop specific antifibrotic therapies towards clinical translation. In order to examine the translatability of potential therapeutic targets, we systematically integrated multiple public or in-house human, mouse and rat datasets, each pertaining to liver fibrosis of a different etiology. A total of 61 significantly upregulated genes in aHSCs were identified specific to liver fibrosis independent of etiology. Our analysis prioritized *LARP6*, *AEBP1* and *PRRX1* because they have been linked to liver fibrogenesis. Pathway enrichment analysis of the 61 genes revealed that, despite the diverse causative factors of liver fibrosis, ECM organization and integrin cell surface interactions are the canonical and common pathological pathways involved in liver fibrosis. Gene regulatory network analysis of the 61 genes allowed the identification of *CREB3L1* as a master regulator for fibrosis-associated genes. Overall, this suggests that *CREB3L1* might be a promising novel drug target for liver fibrosis, with established translatability in key rodent models, and modulation of it is likely to result in effective anti-fibrotic activity. TFs have historically been viewed as ‘undruggable’ due to their pleiotropic actions in multiple cell types and lack of defined ligandable pockets^68^. Thanks to recent advances in structural characterization and ligand design strategies, TFs have been directly targeted by multiple enticing modalities including, for example, small-molecule protein–protein interaction inhibitors and targeted protein degradation^68^. *CREB3L1* belongs to the CREB (cAMP response element binding protein) family. Vannam et al.^69^ recently reported the design and characterization of dCBP-1, a potent and selective heterobifunctional degrader of the CREB-binding protein (CBP) that is involved in the transcriptional coactivation of multiple TFs including *CREB3L1*.

Taken together, our findings provide potential targets for the treatment of NASH with fibrosis and helpful clues for better understanding the molecular underpinnings of liver fibrosis onset and progression. Our analysis also sheds light on the common fibrotic gene signatures across human and rodents, and also between human organs, which demonstrate high translatability for further investigation.

## Methods

### Liver tissue collection and sequencing of liver non-parenchymal cells

Physiologically normal human liver tissues were collected from the periphery of liver specimens from donors undergoing surgical resection. In all of these cases, the resections were done in a generous way in order to ascertain removal of all affected tissue. Of the six patients whose tissue we used, three underwent surgery for liver metastasis of rectal adenocarcinoma, and there was one patient each for hepatocellular adenocarcinoma (inflammatory type), hepatolithiasis, and liver hemangioma. All tissue resections were inspected by experts both macroscopically and microscopically to identify the “most healthy” parts, i.e., most distal of any potential lesions. Overall, through the combination of careful histopathological assessment and comparisons to for example data derived from hepatocellular carcinoma, we are confident that our “healthy” tissue is as good a proxy for healthy tissue as is realistically and ethically achievable. Informed consent of the patients was obtained in accordance with institutional guidelines and the local ethics committee in Bern, Switzerland. This study was approved by the regional ethics committee in Basel, Switzerland (Ethikkommission Nordwest- und Zentralschweiz; #2015-391 PB_2017-00323).

Liver tissue was stored at 4 °C and processed within 3 h post resection. Liver non-parenchymal cells (NPCs) were isolated following a two-step protocol of collagenase/EDTA digestion^70^. Briefly, hepatocytes were removed by centrifuging the whole digestion at 50 g for 5 min (pellet removed—hepatocyte fraction, cleaned on Percoll), and the supernatant removed and spun again at 50 g for 5 min to remove the remaining hepatocytes, the supernatant was then pelleted at 300 g for 8 min and used as NPC fraction for downstream sequencing library preparation. Cell viability of the isolated NPCs was estimated by trypan blue exclusion. Samples of 67–89% viable cells from each of the liver samples were subjected to single-cell RNA sequencing using the 10× Genomics Chromium Single Cell 3’ Library & Gel Bead Kit v2 following the manufacturer’s instructions. Approximately 4000 cells were targeted for each sample recovery. Libraries were sequenced on the Illumina HiSeq2500 machine using paired-end sequencing runs.

### Generation of bulk RNA-seq data for both BDL and TAA-induced liver fibrosis rat models

All in vivo animal studies were reviewed and approved by the Institutional Animal Care and Use Committee (IACUC) of Novartis Institutes for BioMedical Research in Shanghai, China (approval #2013-05-01 (BDL model); #2016-03-08 (TAA model)). All experiments were performed in accordance with the relevant guidelines and regulations and in compliance with the ARRIVE guidelines.

To induce hepatic fibrosis, male Sprague Dawley rats (9–12 weeks of age and 380–420 g of weight upon arrival, supplied by Beijing Vital River laboratory animal Co., Ltd.) either underwent surgery of bile duct ligation (BDL) or were treated with thioacetamide (TAA). In the BDL model, the bile ducts of Sprague Dawley rats were ligated after 12 h of fasting and water deprivation. Rat liver samples were collected from three groups of rats at week 1, 2 and 5 after BDL surgery. Three control groups of rats underwent sham operation, including bile duct mobilization, but without BDL. Three biological replicates were used for each group. In the TAA model, rat liver samples were collected from five groups of rats at week 1, 2, 4, 8 and 13 after TAA (300 mg/kg) administration three times per week while five control groups received the same volume of 0.9% normal saline. Four biological replicates were used for each group.

Frozen rat liver tissue (~ 2 mm cube) was lysed and homogenized using Trizol reagent in a Qiagen TissueLyser II. After phase separation of the homogenate, the upper aqueous phase was subject to total RNA purification using Qiagen RNeasy Mini spin column with extensive on-column DNase I digestion. Purified total RNA samples were analyzed by Agilent Bioanalyzer RNA 6000 Nano assay and quantified by Qubit RNA assay. For construction of sequencing libraries, 1 ug of total RNA (RIN > 8) was subjected to Illumina TruSeq Stranded Total RNA Sample Prep kit with Ribo-Zero Gold. Libraries were analyzed by Bioanalyzer High Sensitivity DNA assay and quantified by Qubit DNA assay. Sequencing was performed by Illumina NextSeq 500 or HiSeq 1500.

### Single-cell RNA-seq data analysis

Library demultiplexing, fastq file generation, read alignment and unique molecular identifier (UMI) quantification were performed using the Cell Ranger Single Cell Software Suite v3.1.0 (10× Genomics, Inc.) against the 10× Genomics pre-built GRCh38.93 human reference genome. Data processing and visualization were performed with the Seurat package v3.1.5^32^ in R v3.6.1 (R Core Team, 2019). The initial dataset contained 175,429 cells, namely, 17,810 cells that we profiled and 157,619 cells obtained from a public dataset^22^ for 33,538 genes. Since in our study we revolve around all liver non-parenchymal cells with a focus on hepatic stellate cells, for the public dataset we only included the seven donors with both leucocytes (CD45^+^) and other non-parenchymal cells (CD45^−^) profiled. Outlier cells were excluded from the downstream analyses using the *isOutlier* function from the scater R package. Moving along, we only kept genes expressed in at least 10 cells and cells with at least 200 genes expressed. Raw UMI counts were log-normalized and then 2000 highly variable genes were identified with the Seurat function *FindVariableFeatures*. Scaling was performed to regress out confounders, i.e., total UMI count, percent of mitochondrial reads and cell cycle score. Next, principal component analysis was performed using the variable genes for dimensionality reduction using the Seurat function *RunPCA*. Clusters were identified with the Seurat function *FindClusters* using the shared nearest neighbor modularity optimization based on the first 12 PCs with a clustering resolution set to 0.4. This method resulted in 20 initial clusters. Of the parameters we tried, most produced a similar UMAP clustering, but 12 PCs generated the best separation between different cell types. For each cell type, marker genes were identified using the Seurat function *FindAllMarkers* with the Wilcoxon rank-sum test. The *FindAllMarkers* function was also used to identify differentially expressed genes in activated HSCs compared with quiescent HSCs, and also in activated PSCs compared with quiescent PSCs. Scaled expression data for marker genes were used to create the heatmap.

### Automated annotation of cells with SingleR

We compared our manual annotations of clusters based on known cell-type-specific marker genes to that produced through automated classification using SingleR^33^, which leverages reference transcriptomic datasets (Human Primary Cell Atlas, in the case of our study) of pure cell types to infer the cell of origin of each of the single cells independently.

### ECM score calculation

The extracellular matrix (ECM) score for each mesenchymal cell was calculated based on the normalized expression values of matrisome genes using the method as previously described^71^.

### Intercellular communication analysis with CellPhoneDB

To infer ligand-receptor interactions present within the liver, we applied CellPhoneDB (v2.1.4), which determines cellular crosstalk based on significant enrichment of reciprocal expression of ligands and receptors between annotated cell populations^35^. In brief, we derived potential ligand-receptor interactions on the basis of the expression of a receptor by one cell population and a ligand by another; as input to this algorithm, we used all cells, and we considered only ligands and receptors expressed in more than 5% of the cells in any given cell population. Cell population-specific interactions were identified as follows: (1) randomly permuting the cluster labels of all cells 1,000 times and determining the mean of the average receptor expression of a cell population and the average ligand expression of the interacting cell population, thus generating a null distribution for each ligand-receptor pair in each pairwise comparison between cell populations; (2) calculating the proportion of these means that were ‘as or more extreme’ than the actual mean, thus obtaining a *P* value for the likelihood of cell population specificity for a given ligand-receptor pair; (3) prioritizing interactions that displayed specificity to cell populations interacting within the liver.

### Gene regulatory network (regulon) analysis

SCENIC is a computational tool that infers regulons by analyzing the co-expression of transcription factors and their putative target genes characterized by enrichment of corresponding transcription factor-binding motifs in their regulatory regions^48^. Regulatory network and regulon activity analysis were conducted on the mouse (mm10) and human (hg38) datasets separately using pySCENIC (v0.10.2) with default parameters. Normalized log counts were used as input to identify co-expression modules by the GRNBoost2 algorithm. Following which, regulons were derived by identifying the direct-binding TF target genes while pruning others based on motif enrichment around transcription start site (TSS) with cisTarget databases. Using aucell, the regulon activity score (RAS) was measured as the area under the recovery curve (AUC). Scaled cell type-averaged RAS > 0.7 was regarded as significant. Overall, we identified 176, 340 and 532 active regulons for human mesenchymal cells, mouse mesenchymal cells and human pancreatic cells, respectively.

### RNA velocity analysis

RNA velocity analysis was performed as previously described using the velocyto.py python package for annotating transcripts as spliced or unspliced^72^, followed by the scVelo pipeline (v0.2.2)^73^. Briefly, transcripts are marked as either spliced or unspliced based on the presence or absence of intronic regions in the transcript. For each gene, a simple model of RNA dynamics is then fit to the data. Finally, the RNA velocity is estimated for each cell by looking for over- or underrepresentation of spliced to unspliced ratios. RNA velocity is visualized on a UMAP plot, with vector fields representing the averaged velocity of nearby cells.

### Bulk RNA-seq data analysis

The exon quantification pipeline (EQP)^74^, a tool based on Bowtie 2.0, was used to generate count-based gene expression estimates, determined by an alignment of the reads to the human genome (GRCh38.p10, Ensembl 90, August 2017). Depending on the context, either CPM (counts per million) or TPM (transcripts per million) was calculated to represent gene expression levels. Differential gene expression analysis was performed using the DESeq2 R package (v1.22.2)^75^ in R (v3.6.1) with $design = \sim Condition$ for pairwise differential expression analysis, and $full\_model = \sim Condition + Timepoint + Condition:Timepoint$ and $reduced\_model = \sim Condition + Timepoint$ for time-course differential expression analysis. P-values for each gene were adjusted using the *p.adjust()* function in R with the Benjamini–Hochberg method. Genes with an adjusted *P* < 0.05 were deemed to be significant.

### Deconvolution of publicly available human NASH bulk RNA-seq data

Computational deconvolution of bulk RNA-seq data was performed using MuSiC^76^ with the following parameters: iter.max = 1000, nu = 1e-10, eps = 0.01, and normalize = F.

### Orthologous gene sets

To evaluate the translatability of potential therapeutic targets across species, only genes with a one-to-one orthologous relationship across the species investigated (i.e., human, mouse and rat) were considered. All possible homolog pairs between human, mouse and rat were first downloaded from Ensembl release 93 via BioMart. We then only kept 15,252 genes with a one-to-one correspondence between any species pairs (Supplementary Table 6).

### Pathway enrichment analysis for differentially expressed genes

The clusterProfiler^77^ and ReactomePA^78^ R packages were used to perform Reactome pathway enrichment analysis for the 61 liver fibrosis-specific genes independent of causative factors. Fisher’s exact test followed by the Benjamini–Hochberg correction was performed for statistical analysis, and an adjusted *P* < 0.05 was set as the cutoff criterion.

## Supplementary Information

Supplementary Information 1.

Supplementary Information 2.

## Data Availability

Single-cell RNA-seq data of human liver are available from the ArrayExpress database under Accession Number E-MTAB-10553. Bulk RNA-seq data of thioacetamide (TAA)- and bile duct ligation (BDL)-induced liver fibrosis rat models can be accessed with ArrayExpress Accession Numbers E-MTAB-10547 and E-MTAB-10546, respectively. Publicly available human liver and human pancreas scRNA-seq datasets were obtained from the GEO database [GSE136103 and GSE84133]. Publicly available human NASH bulk RNA-seq data were retrieved from the NCBI Bioproject database under Accession PRJNA512027. Publicly available single-cell and bulk RNA-seq data of liver fibrosis mouse models were downloaded from the GEO database [GSE137720 and GSE116987].

## Code availability

No new algorithms were developed for this manuscript. R scripts enabling the main steps of the analysis are available from the authors upon request.
