# Supplementary material for: Single-cell and bulk transcriptomics of the liver reveals potential targets of NASH with fibrosis
Source: Sci Rep. 2021 Sep 29;11:19396. doi: 10.1038/s41598-021-98806-y (PMC8481490; doi:10.1038/s41598-021-98806-y)
Supplement: Supplementary file 1 — Supplementary Information 1. [file 41598_2021_98806_MOESM1_ESM.docx]

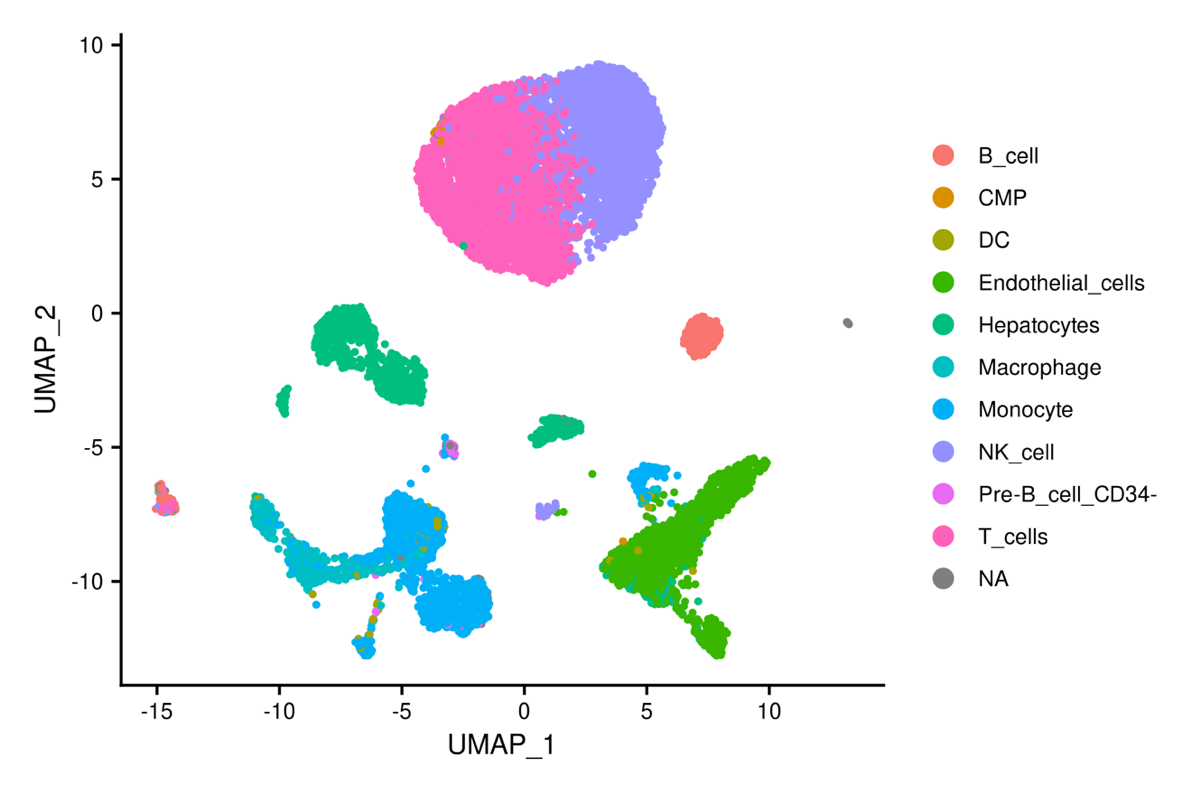


**Supplementary Fig. 1.** Automated cell type annotation by SingleR. UMAP embedding of all cells profiled in this study colored by automatically generated reference-based annotations from Human Primary Cell Atlas.


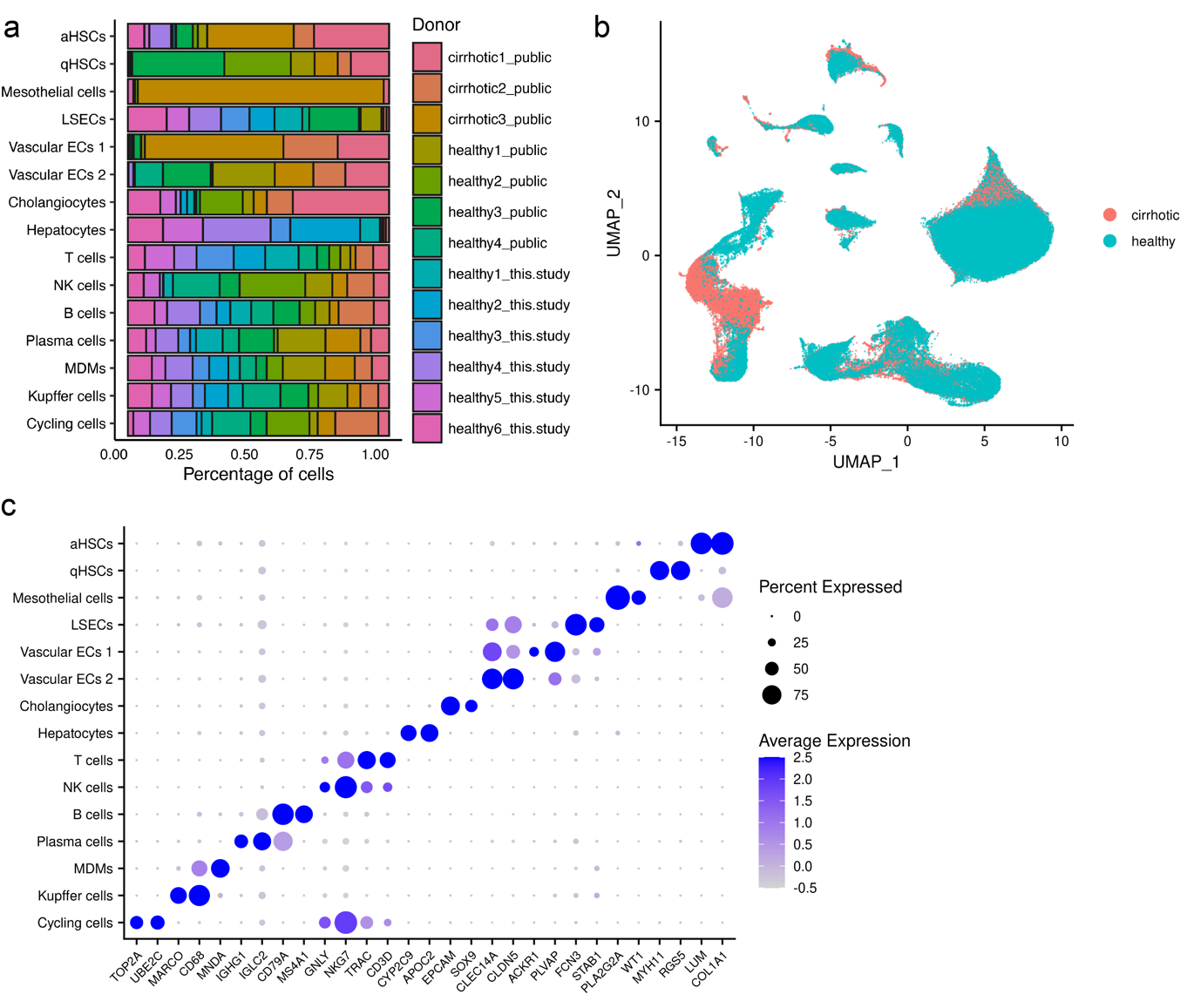


**Supplementary Fig. 2.** Analysis of integrated human liver scRNA-seq data. (**A**) Barplot representing the relative contribution of cells from each donor for each cell type. q/a HSCs, quiescent/activated hepatic stellate cells; LSECs, liver sinusoidal endothelial cells; Vascular ECs, vascular endothelial cells; MDMs, monocyte-derived macrophages; NK cells, natural killer cells. (**B**) UMAP visualization of single cells. Each point depicts a single cell, colored according to disease stage. (**C**) Dotplot displaying select differentially expressed genes for each cell type identified. Size of the dot represents proportion of the cell population that expresses each gene. Color indicates level of expression.


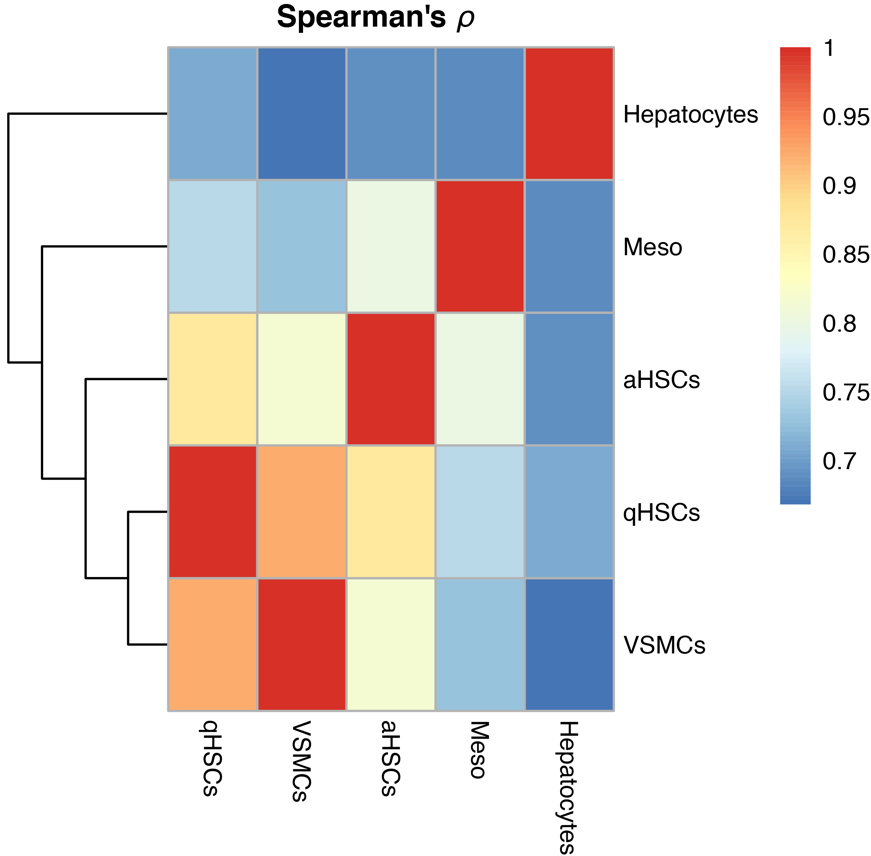


**Supplementary Fig. 3.** Correlations of gene-expression levels between cell types. The heatmap of the pairwise Spearman’s correlation coefficient (*ρ*) is based on genes with their expression averaged across cells for each cell type. It represents the degree of similarity of gene-expression profiles between cell types. Meso, mesothelial cells; q/a HSCs, quiescent/activated hepatic stellate cells; VSMCs, vascular smooth muscle cells.

**
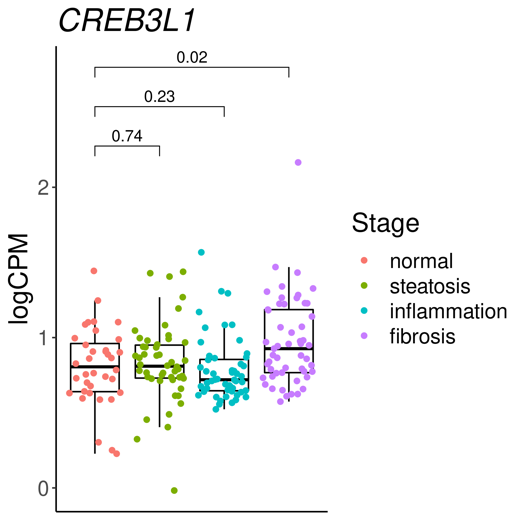
**

**Supplementary Fig. 4.** Expression of *CREB3L1* across the nonalcoholic fatty liver disease spectrum. RNA-seq data of the 191 liver samples (normal, 36; steatosis, 50; inflammation, 52; fibrosis, 53) obtained from Gerhard et al. (2018) were reanalyzed. Mann–Whitney *U* tests (two-sided) were performed for statistical pairwise comparisons. logCPM, log-transformed Counts per Million.
